# Role of bone morphogenetic protein-2 in osteogenic differentiation of mesenchymal stem cells

**DOI:** 10.3892/mmr.2015.3954

**Published:** 2015-06-18

**Authors:** JIAN SUN, JIEYUN LI, CHICHI LI, YOUCHENG YU

**Affiliations:** Department of Dentistry, Zhongshan Hospital, Fudan University, Shanghai 200032, P.R. China

**Keywords:** bone morphogenetic protein-2, bone mesenchymal stem cells, osteogenesis, alkaline phosphatase, osteocalcin

## Abstract

Bone mesenchymal stem cells (BMSCs) have been an area of interest in biomedical research and tissue engineering due to their diverse differentiation abilities. In osteogenesis, bone morphogenetic proteins (BMPs), particularly BMP-2, are important. However, the effect of BMP-2 on the osteogenetic capacity of BMSCs remains to be fully elucidated. In the present study, primary rat BMSCs were infected with a recombinant lentivirus carrying the BMP-2 gene (Lenti-BMP-2), and the effects of BMP-2 on the activity of alkaline phosphatase (ALP) on days 3, 7, 14 and 21, and on mineralization on day 21 were evaluated. In addition, the adhesive ability of BMP-2-overexpressed BMSCs was detected using an adhesion assay. Following forced expression of BMP-2 in the BMSCs, the levels of osteogenic genes, including osteopontin (OPN), osteocalcin (OC) and collagen type I (Col-I), were detected and the nuclear accumulation of Runt-related transcription factor (Runx)-2 and phosphorylated small mothers against decapentaplegic (p-Smad) 1/5/8 was also evaluated. The results demonstrated that the rat BMSCs had been isolated, cultured and passaged from Sprague-Dawley rat bone marrow successfully, and the third-generation BMSCs were identified using flow cytometry with CD29 staining. The osteogenetic phenotype of the BMSCs, expressing ALP and osteocalcin, was significantly induced by BMP-2, and the proliferation of the BMSCs was enhanced by BMP-2. Furthermore, the adhesive potential of the BMP-2-overexpressed BMSCs was increased, the expression levels of OPN, OCN and Col-Ie osteogenetic factors were upregulated and the nuclear accumulation of Runx-2 and p-Smads1/5/8 were increased significantly. These data suggested that BMP-2 may facilitate the osteogenetic differentiation of rat BMSCs and provide a favorable cell resource for tissue engineering.

## Introduction

With the progress of tissue engineering, bone mesenchymal stem cells (BMSCs) are frequently investigated and are being increasingly accepted as useful tools for bone tissue engineering due to their potential to differentiate into a variety of lineages (1). For example, BMSCs have been used successfully in reconstruction of the skull, based on scientific evidence that BMSCs can be induced into osteoblasts (2). In oral implantation, it has been suggested that the osteoblasts adhering at the implant surface originate from the bone marrow and migrate to the location of the implant (3). At present, the osteogenic lineage differentiation of BMSCs may be the most well described, and bone morphogenetic proteins (BMPs) may be the best-characterized cytokines driving osteogenic differentiation (4).

BMP is a member of the transforming growth factor-β superfamily. It is a multifunctional acidic polypeptide, which is predominantly synthesized and secreted by osteoblasts (5). At present, >20 subtypes of BMPs have been identified (6). Among these, BMP-2 is an important regulating factor in osteogenesis (7). BMP-2 is a polypeptide growth factor containing 396 amino acids, the function of which induces undifferentiated mesenchymal cells into cartilage and bone tissues (8). BMP-2 may provide a basis for a tissue-engineered bone construct, which is compatible with the growing craniofacial skeleton, but without the morbidities associated with distant graft harvest (5). However, the induced effects of BMP-2 on osteogenesis remain to be elucidated and require further investigations.

In the present study, BMP-2 was overexpressed in BMSCs through lentivirus vectors to determine the effects of BMP-2 on BMSCs osteogenetic differentiation and to improvie understanding of the molecular basis of BMP-2-mediated osteogenesis.

## Materials and methods

### BMSC primary culture and identification

A total of 32 Sprague-Dawley (SD) rats, aged 18 weeks and weighing ~300 g, were provided by the Animal Experiments Center, Zhongshan Hospital, Fudan University (Shanghai, China). The femur and tibia were obtained and soft tissues were removed prior to immersion in low-glucose Dulbecco's modified Eagle's medium (L-DMEM, cat. no. 11885-092; Gibco Life Technologies, Carlsbad, CA, USA). Rats were housed in groups of three under controlled temperature (22±2°C), relative humidity (55±10%), 12-h light/dark cycle (7:00 a.m. to 7:00 p.m.) and provided with food and water *ad libitum*. Carbon dioxide (CO_2_) inhalation was used as a method of euthanasia for rats. Rats in the euthanasia chamber were exposed to 100% CO_2_ for 7 min. All animal experiments were performed according to the EU directives of 2010 on the protection of animals used for scientific purposes. The marrow cavities were washed with L-DMEM and the resulting solution, containing the bone marrow, was collected. Following collection, the BMSCs were cultured with fresh L-DMEM containing 10% fetal bovine serum (FBS; cat. no. 10099-141; Gibco Life Technologies) in the incubator (NAPCO 5410; NAPCO, Chicago, IL, USA) at 37°C and 5% CO_2_. At 48 h post-seeding, the culture medium was replaced for the first time, and these cells were termed the first generation (P1) cells. The cell morphology and growing conditions were visualized using an inverted microscope (BX-40; Olympus, Hamburg, Germany).

### Flow cytometric analysis

The fluorescein isothiocyanate (FITC)-conjugated CD29 BMSC biomarker (Abcam, Cambridge, MA, USA) was analyzed using a BD™ Flow Cytometer (BD Biosciences, Franklin Lakes, NJ, USA) using BD CellQuest Pro software version 5.1 (BD Biosciences) to identify the harvested cells. The cells were then incubated with 10 *µ*l CD90 primary anti-rat polyclonal antibodies (FITC-conjugated; 1:1,000 dilution; Vector Laboratories, Inc., Burlingame, CA, USA) and with a negative control of the same type.

### Cell counting kit-8 (CCK-8) analysis

The P1 cells of the BMSCs were seeded into a 96-well plate at a density of 1×10^3^ and six repeated wells were arranged as a blank control. CCK-8 solution (Dojindo Molecular Technologies, Inc., Kumamoto, Japan) was added to each well (10 *µ*l per well), according to the manufacturer's instructions. The optical density (OD) value was measured at a wavelength of 450 nm, with the results presented as the mean.

### Overexpression of BMP-2 in BMSCs

Lentivirus vectors containing the BMP-2 and enhanced green fluorescent protein (EGFP) genes were constructed with the assistance of Shanghai Genechem (Shanghai, China), and were expanded by culture of HT1080. The HT1080 cell line was used for lentivirus vector production (Shanghai BioHermes Bio-Pharmaceutical Technology, Co., Ltd., Shanghai, China). The total cells were divided into three groups. The BMP-2 group was composed of cells transduced with Lenti-BMP-2. The cells in the mock group were transduced with blank lentivirus vectors, while the cells in the control group remained untransduced. The P3 BMSCs were infected at a multiple of infection (MOI) of 25, transferred to fresh medium and maintained in an incubator at 37°C and 5% CO_2_. The effects of transduction and growing conditions were observed using fluorescence microscopy (Olympus BX61; Olympus).

### RNA extraction and reverse transcription-quantitative polymerase chain reaction (RT-qPCR)

Total RNA was extracted from each group using TRIzol (Invitrogen Life Technologies, Carslbad, CA, USA) and purified, according to the manufacture's instructions. RT of the mRNA was performed using a Reverse Transcription kit (Takara, Bio., Inc., Shiga, Japan), according to the manufacturer's instructions. The expression level of genes were normalized to β-actin. The primers used for amplification were designed, according to the nucleic acid sequences in the Gene bank and are listed in Table I. qPCR was performed using SYBR Green I (Takara, Bio, Inc.), according to the manufacturer's instructions. The amplification procedure used the following program: Denaturation at 95°C for 5 min, 95°C for 30 sec, 55°C for 30 sec, 72°C for 1 min (repeated for 30 cycles) and extension at 72°C for 8 min. The total volume of 5 *µ*l qPCR product was separated onto 1.5% agarose gels for electrophoresis. The net index (NI) of the stripes' gray scale was analyzed using ScanImage software version 4.1 (BD Biosciences) and were compared with that of β-actin, the internal reference. All experiments were performed in triplicate.

### Western blot analysis

The cells from each group were washed with phosphate-buffered saline (PBS) and lysis buffer (Qiagen, Valencia, CA, USA) pre-cooled on ice, and eight samples were selected from each group. Proteins (3 *µ*g/*µ*l) were separated onto 10% SDS-PAGE polyacrylamide gels (Invitrogen Life Technologies) for electrophoresis. The nitrocellulose membranes (Bio-Rad Laboratories, Inc., Hercules, CA, USA), used as the transference medium, were blocked in non-fat milk in Tris-buffered saline with Tween 20 (TBST) for 3 h, and hybridized with primary antibodies for BMP-2 (rabbit anti-rat BMP-2 polyclonal antibody; 1:200; EMD Millipore, Billerica, MA, USA; cat. no. P12643), osteopontin (mouse anti-rat OPN monoclonal antibody; 1:1,000; Santa Cruz Biotechnology, Inc., Dallas, TX, USA; cat. no. sc-21742), osteocalcin (mouse anti-rat OCN monoclonal antibody; 1:1,000, Santa Cruz Biotechnology, Inc.; cat. no. sc-365797) and collagen type I (goat anti-rat COL1 polyclonal antibody; 1:500; Santa Cruz Biotechnology, Inc.; cat. no. sc-166865). overnight at 4°C. The membranes were washed with PBS three times and were incubated with secondary antibodies (Jackson Immunoresearch Laboratories, Inc., West Grove, PA, USA) for 2 h at room temperature. The protein bands were visualized by enhanced chemiluminescence detection reagents (Applygen Technologies Inc., Beijing, China) subsequent to washing in PBS three times. The NI of the gray scale of the bands were assessed, with GAPDH used as the internal reference. All experiments were performed in triplicate.

### Alkaline phosphatase (ALP) activity

To determine whether the early osteogenic differentiation of BMSCs was induced by BMP-2, ALP staining was performed, as previously reported (9). Following 3, 7, 14 and 21 days of culture, the cells were fixed in 10% formalin, washed with PBS and then incubated in staining solution, containing a mixture of 0.02% 5-bromo-4-chloro-3-indolyl phosphate (BCIP; Santa Cruz Biotechnology, Inc.) and 0.03% nitro blue tetrazolium (NBT; Santa Cruz Biotechnology, Inc.) in 0.1 M TBS, which was added into 5 ml AP buffer (100 mM Tris-HCl, 100 mM NaCl, 5 mM MgCl_2_ and 0.05% Tween 20, pH 9.5) and incubated for 1 h at room temperature. Furthermore, the activity of ALP and protein content of the three groups were measured on days 3, 7, 14 and 21. The cells lysates were prepared, and the activity of ALP in the lysates was determined using a Lab-Assay-ALP colorimetric assay kit (Wako Pure Chemicals, Osaka, Japan), according to the manufacturer's instructions. The total protein concentrations were determined using a commercial BCA Protein Assay kit (Beyotime Institute of Biotechnology, Shanghai, China). The activity of ALP was calculated as nmol/h phosphorylated Nitrophenol (p-NP) release and was further normalized to the cell protein content.

### Alizarin red staining

The BMSCs with or without BMP-2 were dissociated and passaged using 0.25% trypsin (Sigma-Aldrich) to produce adherent cell slices. When the cells reached confluence at 80% on the cover slips, the medium was substituted with osteogenetic induction solution (Shanghai Sangon Biotech, Co., Ltd., Shanghai, China) containing 0.1 *µ*mol/l dexamethasone, 50 mg/l ascorbic acid and 10 mmol/l β-glycerophosphate, and cultured at 37°C and 5% CO_2_. The medium was replaced every 3 days. On the 21st day, the cells were fixed with 95% ethanol for 10 min, and incubated in 2% Alizarin red staining solution (Sigma-Aldrich) for 5 min. The staining reactions were terminated by washing the cells with PBS. Calcification deposits were identified in the matrix, which appeared bright red under light microscopy (Olympus CX23; Olympus) and images of the cells were captured. The calcification was quantified by determining the densities and areas of Alizarin red staining using an image analysis program (Multi Gauge V3.0 software; Fujifilm, Tokyo, Japan).

### Adherence assay

The cells were routinely passaged and counted, and then diluted in serum-free medium to a density of 2×10^5^/ml. Subsequently, 100 *µ*l of the cell suspensions were seeded into each well in a 96-well plate, which had been previously wrapped with fibronectin. After 3 h incubation at room temperature, the medium was removed and the non-adherent cells were removed by washing in PBS. The adherent cells in each well were fixed using 50 *µ*l 4% paraformaldehyde for 10 min and stained with 50 *µ*l gentian violet staining solution (Invitrogen Life Technologies) for 15 min at room temperature. Following staining, the cells were counted under a microscope (Olympus). After staining with 100 *µ*l gentian violet in each well, the cells were maintained at 37°C and, after 20, 40 and 60 min, the OD value was measured using microplate spectrophotometer (Shimadzu UV-2450; Shimadzu Corp., Kyoto Japan) at a wavelength of 585 nm.

### Subcellular fractionation

Cellular fractionation was performed using NE-PER Nuclear and Cytoplasmic Extraction reagents (Pierce Biotechnology, Inc., Rockford, IL, USA), according to the manufacturer's instructions. Subsequently, fractions were processed, as described above for western blotting. The membranes were incubated with either rabbit anti-Runt-related transcription factor (Runx)-2 polyclonal antibody (1:500; Santa Cruz Biotechnology, Inc.; cat. no. sc-10758), or rabbit anti-p-small mothers against decapentaplegicp (p-Smad) 1/5/8 antibody (1:1,000; Cell Signaling Technology, Inc.; cat. no. 9516) overnight at 4°C. The subsequent steps were performed as described above. To normalize the bands, the filters were removed and re-probed with anti-paxillin (1:1,000; BioLegend, Inc., San Diego, CA, USA) and anti-B23/nucleophosmin (Santa Cruz Biotechnology, Inc.) antibodies. The density of the bands were quantified densitometrically. Densitometric quantification of the protein bands was analyzed by TINA software version 2.1 (Raytest Isotopenmessgeräte GmbH, Straubenhardt, Germany).

### Statistical analysis

All data are presented as the mean ± standard error of the mean. Comparisons between groups were analyzed using analysis of variance using SPSS 11.0 software (SPSS, Inc., Chicago, IL, USA). P<0.05 was considered to indicate a statistically significant difference. The P-values were corrected for multiple testing procedures and to control for type I error rates.

## Results

### Morphological characteristics and identification of BMSCs

**Figure 1:**
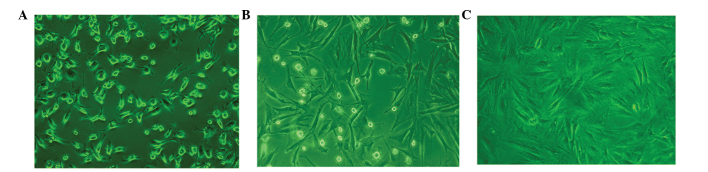
Morphological characteristics of primary cultured BMSCs. (A) In the first 48 h, the cells became adherent and were spindle-shaped or polygonal. (B) BMSCs in the 3rd passage (P3) were consistent in spindle shape. (C) BMSCs in the 20th passage (P20) exhibited no significant difference from those in P3.

**Figure 2:**
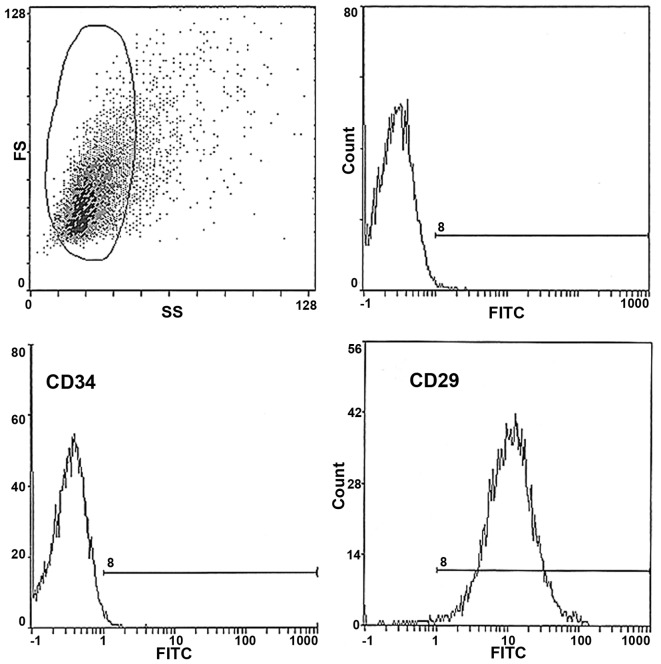
Flow cytometric analysis of the BMSCs. Graph depicting the flow cytometric analysis of the expression of CD29^+^ and CD34^−^ surface markers in the P3 rat BMSCs. BMSC, bone mesencymal stem cell; BMP, bone morphogenetic protein; FS, forward scatter; SS, side scatter; FITC, fluorescein isothiocyanate.

The BMSCs were spherical in shape shortly following seeding, the majority of which were suspended in the medium. After 48 h, the majority became adherent and exhibited a different morphology, predominantly spindle-shaped or polygonal. The nuclei were usually large and located in the middle or margin of the cells. Scattered adherent fibroblast-like cells were observed on the 3rd day of culture, following which the passaged cells became completely adherent within 24 h. The shape of the spindle in the P3 cells was consistent. Following subculture for >20 generations, the cells maintained vigorous growth and amplification, and no obvious difference in the duration required to reach confluence were observed (Fig. 1). In addition, the biomarkers of BMSCs were characterized using flow cytometry (Fig. 2), which revealed >85% of the expanded P3 BMSCs were positive for the surface biomarker of CD29, characteristic of BMSCs. In addition few hematopoietic cells were observed, as indicated by the low level of CD34-expressing cells. These results indicated that the P3 cells were highly purified BMSCs.

### Growth pattern of the BMSCs

**Figure 3:**
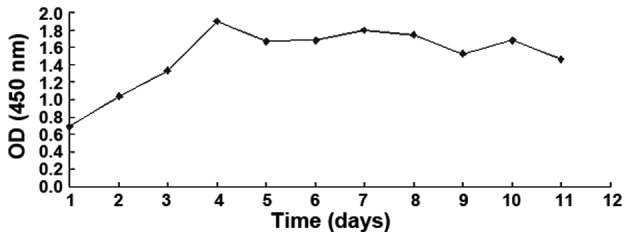
Growth curve of BMSCs. BMSC growth occurred in three phases, the delayed growth phase (days 1–3), logarithmic growth phase (days 3–8) and the growth plateau (days 8–11). BMSC, bone mesenchymal stem cell; BMP, bone morphogenetic protein; OD, optical density.

The growth curve, which was produced according to the OD values of each well (Fig. 3), demonstrated that the BMSCs of regular seeding density had approximately the same growth circle. In the first 2–3 days following seeding, the cells grew relatively slowly, exhibiting a delayed growth phrase. Consequently, the cells then grew rapidly to reach the logarithmic growth phrase. On days 7–8, the total number of cells reached the highest value. Subsequent to the following growth plateau, the rate of cell growth slowed.

### Overexpression of BMP-2 in the BMSCs

**Figure 4:**
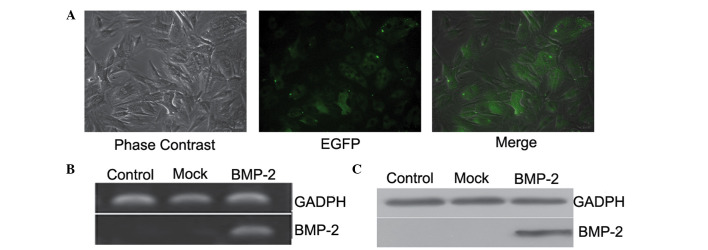
Forced overexpression of BMP-2 in BMSCs. Overexpression of BMP-2 was induced using lentiviral particles containing a construct encoding EGFP. (A) At 72 h post-transduction, EGFP was detected throughout the cytoplasm. Reverse transcription-quantitative polymerase chain reaction and western blot analysis revealed that the (B) mRNA and the (C) protein levels of BMP-2 were significantly increased in the BMP-2-transduced BMSCs, compared with the other cells. Mock, blank lentivirus vector; BMSC, bone mesencymal stem cell; BMP, bone morphogenetic protein; EGFP, enhanced green fluorescent protein.

Overexpression of BMP-2 in the BMSCs was induced using lentiviral particles containing a construct, which encoded EGFP. At 72 h post-transduction, green fluorescence was detected throughout the cytoplasm, in which the transduction efficiency was >90% (Fig. 4A). The RT-qPCR analysis demonstrated that, compared with the mock and control cell groups, the mRNA (Fig. 4B) and protein (Fig. 4C) levels of BMP-2 were significantly increased in the BMP-2-transduced BMSCs, which further confirmed that BMP-2, the target gene, had been successfully transduced into the BMSCs and was expressed efficiently in the cells.

### ALP activity in the BMP-2-induced BMSCs

**Figure 5:**
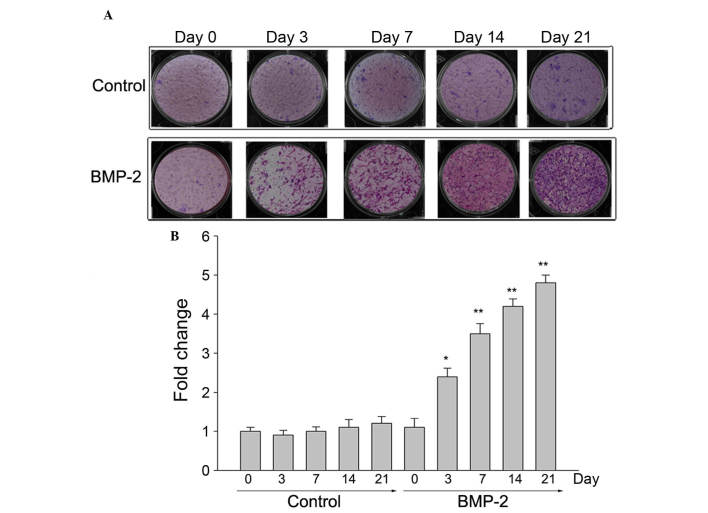
Expression of ALP and activity of BMSCs in the monolayer culture. The expression of ALP was increased in the BMSCs transduced with BMP-2 lentiviral particles, as assessed by (A) ALP staining and (B) quantification, between days 3 and 21. Data are presented as the mean ± standard deviation. *P<0.05 and ^**^P<0.01, compared with the control. BMSC, bone mesencymal stem cell; BMP, bone morphogenetic protein.

The effects of overexpression of BMP-2 on the osteogenic differentiation of BMSCs were examined by examining the expression of ALP, an early osteogenic marker, at indicated time-points (Fig. 5A). Compared with that detected in control BMSCs, the expression of ALP in the BMP-2-transduced BMSCs increased gradually between 2.4-and 4.8-fold between day 3 and 21 (Fig. 5B).

### Extracellular matrix mineralization

**Figure 6:**
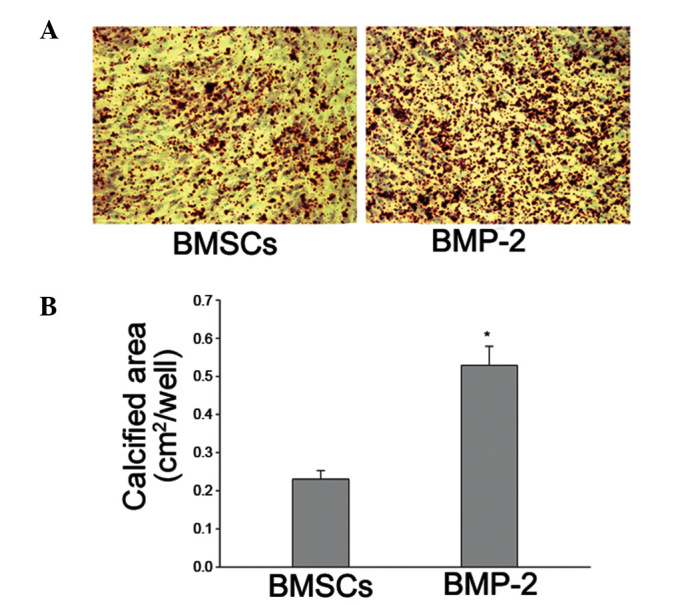
Mineral formation of BMSCs in the monolayer culture. BMSCs reached a confluence of 80% of the cover slips, the medium was then replaced with osteogenetic induction solution. On day 21, compared with the regular BMSCs, mineralization was increased in the BMSCs transduced with BMP-2, as assessed using (A) alizarin red staining in light micrographs and (B) quantification All data are presented as the mean ± standard deviation. *P<0.05, compared with the control BMSCs. BMSC, bone mesenchymal stem cell; BMP, bone morphogenetic protein.

The osteogenic effects of BMP-2 were further characterized by examining the mineralization of the extracellular matrix through Alizarin red staining. As shown in Fig. 6, the extent of mineralization, indicated by the quantity of orange mineralized nodules and calcification areas, was increased 2.3-fold in the BMSCs transduced with the BMP-2 lentiviral particles, compared with the control BMSCs, after 21 days of culture.

### Adherence improvement by BMP-2 in BMSCs

**Figure 7:**
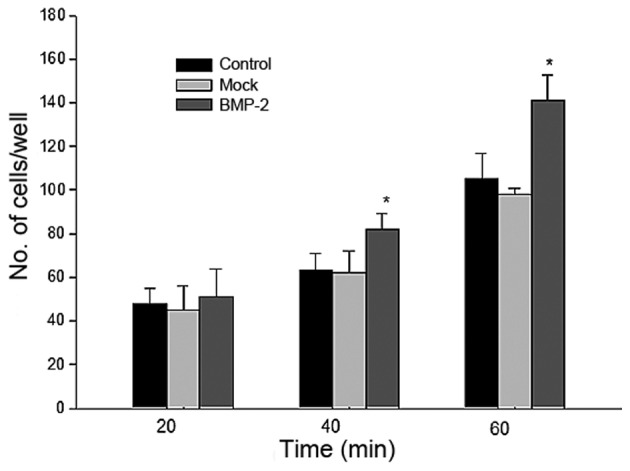
Adherence ability of BMSCs in the monolayer culture. The adherence abilities of the BMSCs transduced with BMP-2 increased with increasing duration. All data are presented as the mean ± standard deviation. *P<0.05, compared with the control BMSCs. BMSC, bone mesenchymal stem cell; BMP, bone morphogenetic protein.

As it is understood that the growth and differentiation of several types of cell, particularly stem cells, is regulated by cell-cell and cell-matrix interactions (10), the adhesive ability of the BMP-2-infected BMSCs was evaluated. The result of the adherence assay revealed that the adhesive ability of the BMSCs significantly improved with time following BMP-2 transduction (Fig. 7).

### Expression and nuclear accumulation of osteogenic markers promoted by BMP-2

**Figure 8:**
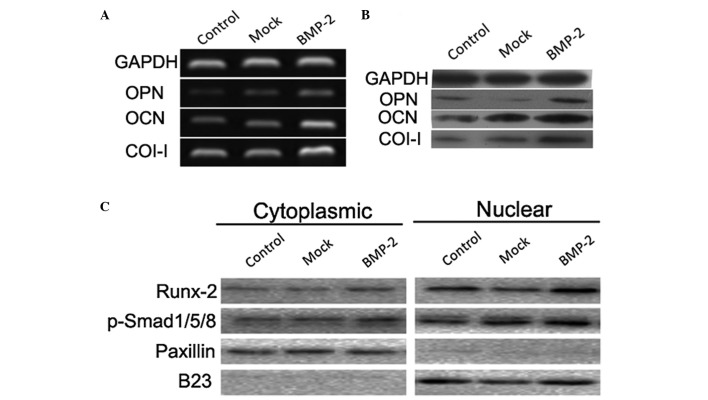
Expression of osteogenic markers promoted by BMP-2. Reverse transcription-quantitatiave polymerase chain reaction and western blot analysis revealed that the (A) mRNA and (B) protein levels of OPN, OCN and COL-I were significantly increased in the BPM-2-transduced BMSCs, compared with the other groups 48 h after lentiviral transduction. (C) Cellular fractionations were performed using NE-PER nuclear and cytoplasmic extraction reagents. Proteins (20 *µ*g) from each sample were subjected to SDS-PAGE, transferred to a polyynilidine difluoride membrane and probed with goat anti-Runx-2 antibody or rabbit anti-phospho-Smad 1/5/8 antibody. The filters were then removed and re-probed with mouse anti-paxillin and rabbit anti-B23 to produce an equal quantity of loading. Mock, blank lentivrus vector; BMP, bone morphogenetic protein; Col-I, collagen type I; OPN, osteopontin; OCN, osteocalcin.

The mRNA and protein levels of OPN, OCN and Col-I in the BMSCs were significantly higher in the BMP-2 group, compared with those in the mock and control groups (Fig. 8A and B). In addition, western blot analysis of the cytoplasmic and nuclear fractions demonstrated that the nuclear accumulations of Runx-2 and p-Smads1/5/8 were significantly increased following BMP-2 transduction into the BMSCs, compared with the control and mock cells (Fig. 8C).

## Discussion

Advances in molecular biology have coalesced with an improved understanding of craniofacial biology to enable what has been termed generative craniofacial surgery (11) Due to various advantages, the use of BMSCs as seeding cells is widespread in investigations of gene therapy, cell substitution therapy and tissue engineering (12–14). In the present study, BMSCs were successfully isolated and purified from an SD rat, based on the combination of morphological characteristics and flow cytometeric analysis using a BMSC-specific marker. This provided an experimental basis for the subsequent experiments.

During the differentiation of BMSCs towards an osteoblastic lineage, several hormones and cytokines are involved and regulate osteogenesis (15). Among these molecules, BMPs are important cytokines in modulating osteogenesis (16,17). Reports have revealed that the procedure of BMP undergoing osteogenetic induction can be divided into four steps: The tendency period, differentiation period, bone formation period and remodeling period. During the whole procedure, BMP-2 is considered to be one of the most active induction factors (13). At present, BMP-2 has demonstrated significant promise as a clinically useful osteo-inductive agent, however, key questions require answering prior to the use of this protein on a large scale as an adjunct to craniofacial surgery (18). Whether BMSC differentiation towards osteoblasts is induced directly by BMP-2 remains to be fully elucidated; and previous findings have demonstrated that BMP-2 either promotes or inhibits osteogenesis (19). As BMP-2 may facilitate a 'paradigm shift', in which molecular techniques reduce the morbidity and mortality rates of craniofacial interventions, while simultaneously enhancing the effectiveness of these procedures, the present study transduced BMP-2 into BMSCs. This was performed using lentiviral vectors, based on evidence that lentiviral vectors are superior to other vectors, including adenoviral vectors, and are considered the ideal vector of gene therapy (20,21). BMP-2 is also critically involved in mediating the condensation of mesenchymal cells and directly differentiating along an osteoblastic phenotype (22). Owing to local stimulation of BMP-2, BMSCs gather through chemotaxis and differentiated into cartilage and bone tissues (23–25). In the present study, forced expression of BMP-2 in the BMSCs not only induced ALP activity and promoted the formation of calcium nodules, but also improved the adherence of BMSCs. BMP-2 also mediated the osteogenic markers, ALP, OCN and Col-I. These results indicated that the BMP-2-transduced BMSCs were in a functional status in the process of osteogenetic differentiation (26,27), as Col-I is secreted by mature osteoblasts (28). In addition, the present study demonstrated that the nuclear accumulation of Runx-2 and hosphor-Smads1/5/8 was significantly increased following BMP-2-transduction of the BMSCs. In the process of osteogenic induction, Runx2 activates and translocates into the nucleus, and acts with Smads through direct binding in a transcriptional activator complex. Runx2 recruits R-Smads to the complex to initiate BMP-responsive gene transcription (29–31).

In conclusion, the results of the present study demonstrated that BMP-2 facilitated the osteogenic differentiation of BMSCs via inducing ALP activity, promoting mineralization, enhancing adherence and mediating the expression and activation of certain associated osteogenic markers. These results indicate the importance of BMP-2 in osteogenesis and, despite several questions remaining unanswered, these results provide experimental evidence supporting advances in craniofacial surgery.

## Figures and Tables

**Table I:** Primers used for amplification.

Gene	Reverse (5′-3′)	Forward (5′-3′)
BMP-2	TTGGAGGAGAAACAAGGTG	AACAATGGCATGATTAGTGG
Col-I	CAGACGGGAGTTTCTCCTCGGACGT	GACCAGGAGGACCAGGAAGTCCACGT
OPN	TGGTTTGCCTTTGCCTGTTCG	ATGGCTTTCATTGGAGTTGCTTG
OCN	GGCGTCCTGGAAGCCAATGTG	GACCAGGAGGACCAGGAAGTCCACGT
β-actin	AGCAGAGAATGGAAAGTCAAA	ATGCTGCTTACATGTCTCGAT

BMP, bone morphogenetic protein; Col-I, collagen type I; OPN, osteopontin; OCN, osteocalcin.
